# Longitudinal Analysis of Rat Gut Microbiome Composition and Fecal Metabolism Markers Following Prolonged Morphine Exposure

**DOI:** 10.3390/biom16030460

**Published:** 2026-03-18

**Authors:** Bianka Micke, Jiri Novotny

**Affiliations:** Department of Physiology, Faculty of Science, Charles University, 12800 Prague, Czech Republic

**Keywords:** morphine, opioids, withdrawal, gut microbiome, fecal metabolome, short-chain fatty acids

## Abstract

This study investigated temporal group-level changes in gut microbiome composition and fecal metabolic markers in Wistar rats following a 10-day administration of morphine. Fecal samples were collected at predefined post-discontinuation time points and analyzed using 16S rRNA gene sequencing and GC×GC-TOF/MS-based metabolomics, with a focus on short-chain fatty acids (SCFAs). Morphine exposure was associated with transient alterations in gut microbiome structure at early post-treatment time points, including changes in alpha diversity and shifts in the relative abundance of major bacterial taxa. Unsupervised multivariate analysis of fecal metabolomic profiles revealed substantial inter-individual variability without persistent global separation between control and morphine-treated groups. Targeted analysis identified transient reductions in the relative signal intensities of selected SCFAs shortly after morphine withdrawal, while no significant differences were observed at later time points. These findings suggest that morphine-associated perturbations of the gut microbiome and fecal metabolome are predominantly time-dependent and tend to diminish during extended post-discontinuation phases.

## 1. Introduction

Morphine remains the gold standard for the management of severe pain due to its potent analgesic and anesthetic properties. Nevertheless, its therapeutic application is constrained by the development of tolerance, physical and psychological dependence, and withdrawal symptoms. While the central nervous system mechanisms underlying these effects have been extensively studied [1,2,3], increasing evidence indicates that chronic opioid exposure also affects peripheral systems, particularly the gastrointestinal tract [4,5,6,7].

The gut microbiome plays a critical role in host physiology by regulating metabolism, maintaining intestinal barrier integrity, and interacting with the immune, endocrine, and nervous systems [8,9,10]. Disruption of microbial homeostasis—commonly referred to as dysbiosis—has been implicated in a wide range of pathological conditions, including obesity, metabolic disorders, inflammatory bowel disease, and immune-mediated diseases [11,12,13]. In parallel, the fecal metabolome reflects the functional output of microbial activity and provides insight into host–microbiome interactions at the metabolic level. Alterations in fecal metabolite composition may therefore contribute to systemic immune dysregulation and inflammation [14,15,16].

The gut microbiome is dominated by two major bacterial phyla, Firmicutes and Bacteroidota (formerly Bacteroidetes), whose relative abundances are often summarized as the Firmicutes/Bacteroidota (F/B) ratio. Although this ratio represents a simplified phylum-level summary of community composition and is widely reported in the literature, its interpretation as a standalone indicator of dysbiosis remains debated [12,17,18]. Alterations in the F/B ratio have been associated with metabolic and inflammatory conditions, suggesting that shifts in this metric may reflect functional changes in the gut ecosystem.

Chronic opioid exposure has been shown to induce gut dysbiosis, increase intestinal permeability, and promote bacterial translocation, resulting in both local and systemic inflammatory responses [4,5,13,19] or to impair the production of antimicrobial peptides in the small intestine, further contributing to the shift in microbial populations [20]. These peripheral effects may interact with central opioid actions via the gut–brain axis, potentially influencing the development of tolerance and the severity of withdrawal symptoms [7,10,21,22]. Animal studies have demonstrated that morphine administration can rapidly induce dysbiosis, characterized by reduced microbial diversity and altered abundance of specific bacterial taxa [4,5]. However, most existing studies have focused on short-term morphine exposure and its immediate effects, typically examining only a few days following drug administration.

In contrast, the long-term trajectories of gut microbiome composition and fecal metabolome following opioid withdrawal remain poorly understood. It is unclear whether morphine-induced dysbiosis persists, resolves spontaneously, or evolves into a distinct microbial state during prolonged withdrawal. Moreover, the temporal dynamics of microbiome and metabolome recovery after opioid discontinuation have not been systematically investigated. This lack of longitudinal data limits our understanding of how opioid exposure affects host–microbiome interactions over time and whether transient or persistent alterations contribute to the pathophysiology of opioid dependence and withdrawal.

The present study aimed to characterize temporal patterns in gut microbiome composition and fecal metabolic markers, with a particular focus on short-chain fatty acids (SCFAs), in Wistar rats subjected to a 10-day intraperitoneal administration of escalating morphine doses. By evaluating predefined post-treatment time points, this approach allows assessment of group-level temporal dynamics following morphine discontinuation.

## 2. Materials and Methods

### 2.1. Animals and Experimental Design

Adult male Wistar rats (weighing approximately 280–300 g) were housed under standard laboratory conditions, with controlled temperature (22 ± 2 °C), humidity (55 ± 10%), and a 12 h light/dark cycle. Animals had ad libitum access to food and water throughout the experiment. Rats were randomly assigned to either a morphine-treated group or a control group. The morphine-treated group received intraperitoneal injections of morphine once daily for 10 consecutive days using an escalating dose regimen ranging from 10 to 50 mg/kg/day (5 mg/kg on days 1–2, 10 mg/kg on days 3–4, 20 mg/kg on days 5–6, 30 mg/kg on days 7–8, 40 mg/kg on day 9, and 50 mg/kg on day 10), while control animals received equivalent volumes of saline. This dosing regimen corresponds to a previously validated protocol that induces dependence while minimizing acute toxicity [23]. All experimental procedures conformed to relevant national and international guidelines and were approved by the Ministry of Education, Youth, and Sports of the Czech Republic (protocol code: MSMT-1479/2019–6; approval date: 25 February 2019).

### 2.2. Sample Collection and Experimental Timeline Design

Fecal samples were collected at predefined time points to capture both short-term and long-term effects of morphine withdrawal. Samples were obtained prior to treatment initiation (baseline), shortly after cessation of morphine administration (24 h after the final morphine dose; day 1), and during prolonged withdrawal at later post-discontinuation time points (days 5, 15, 35, and 60). Immediately after collection, fecal samples were snap-frozen in liquid nitrogen and stored at −80 °C until further analysis. The experimental timeline was designed to enable a longitudinal assessment of gut microbiome and metabolome dynamics following morphine exposure and withdrawal. Fecal samples collected at each predefined post-treatment time point were analyzed as independent observations within treatment groups. The study was designed to evaluate temporal patterns at the group level following morphine discontinuation rather than to track complete within-animal longitudinal trajectories across all time intervals.

Five animals per group were available at each predefined time point. For 16S rRNA gene sequencing, four animals per group per time point were included to enable coverage of multiple withdrawal intervals within available sequencing capacity. For targeted SCFA analysis, five animals per group were analyzed at selected early (day 1) and late (day 60) post-treatment intervals (Supplementary Table S1). No samples were excluded due to sequencing or metabolomic quality control failure; differences in sample size reflect predefined allocation across analytical platforms.

### 2.3. DNA Extraction and 16S rRNA Gene Sequencing

Total bacterial DNA was extracted from fecal samples using the QIAamp Fast DNA Stool Mini Kit (Qiagen, Hilden, Germany; Cat. No. 51604) according to the manufacturer’s instructions, with minor modifications to optimize bacterial cell lysis. DNA quantity and quality were assessed spectrophotometrically. The V3–V4 hypervariable regions of the bacterial 16S rRNA gene were amplified using universal primers and sequenced on an Illumina platform.

Raw paired-end reads were quality filtered using a Q30 threshold, and primer sequences were removed prior to downstream processing. Reads were merged and processed using a standard bioinformatics workflow. Chimeric sequences were identified and removed using the UCHIME algorithm implemented within the clustering pipeline. Operational taxonomic units (OTUs) were clustered at 97% sequence similarity.

A total of 40 fecal samples were included in the final analysis. After quality filtering, chimera removal, and OTU clustering, the median number of high-quality sequences per sample was 16,521 (range: 5236–24,533 reads per sample). To control for variation in sequencing depth, samples were rarefied to a common sequencing depth of 5236 reads per sample prior to alpha diversity analysis. Rarefaction curves demonstrated progressive saturation of observed OTU richness across samples prior to the applied rarefaction depth (Supplementary Figure S1). Taxonomic assignment was performed using the SILVA reference database (version 138).

### 2.4. Microbiome Data Processing and Diversity Analysis

The relative abundances of the dominant bacterial phyla, including Firmicutes and Bacteroidota, were calculated for each sample. The Firmicutes/Bacteroidota (F/B) ratio was included as a descriptive phylum-level summary metric of community composition and was not interpreted as a standalone indicator of dysbiosis.

Alpha diversity was evaluated using complementary diversity metrics to capture different aspects of microbial community structure. Shannon diversity was used to assess overall diversity by integrating both species richness and evenness, while Chao1 was applied as an estimator of species richness with increased sensitivity to rare taxa, and Pielou’s evenness was included to quantify the uniformity of species distribution within samples. The combined use of these indices enabled differentiation between richness-related and evenness-related shifts at the group level.

Between-group differences at each predefined post-treatment time point were evaluated using two-sided Mann–Whitney U tests. To account for multiple comparisons, p-values were adjusted using the Benjamini–Hochberg false discovery rate (FDR) procedure within predefined families of related tests. Both raw p-values and FDR-adjusted q-values are reported.

Beta diversity was calculated using Bray–Curtis dissimilarities on rarefied OTU count data (5236 reads per sample). Community structure was visualized using principal coordinates analysis (PCoA). Overall group- and time-dependent differences in microbial community composition were evaluated using permutational multivariate analysis of variance (PERMANOVA) with fixed effects for group (control vs. morphine), time (days 1, 5, 15, 35, 60), and group × time interaction (999 permutations). Homogeneity of multivariate dispersion was assessed using permutational analysis of multivariate dispersion (PERMDISP) (999 permutations) to ensure that significant PERMANOVA results were not driven by dispersion differences.

Sequence processing, diversity calculations, and downstream statistical analyses were performed using established microbiome analysis workflows implemented in R and Python.

### 2.5. Fecal Metabolomic Analysis

Fecal metabolomic profiling was performed using comprehensive two-dimensional gas chromatography coupled with time-of-flight mass spectrometry (GC×GC-TOF/MS; Pegasus 4D, LECO Corporation) equipped with a cryogenic modulator and a robotic autosampler (MPS, Gerstel; Mülheim an der Ruhr, Germany). Prior to analysis, samples were derivatized using tert-butyldimethylsilyl (TBDMS) reagents following established derivatization protocols for polar metabolite detection [24]. After derivatization, samples were extracted in hexane and subjected to GC×GC-TOF/MS analysis. This analytical platform enables high-resolution separation and detection of a broad range of small polar metabolites present in fecal samples. Metabolites were identified based on mass spectral libraries and retention behavior. For targeted analysis, short-chain fatty acids (SCFAs), including propionic, isobutyric, butyric, valeric, and caproic (hexanoic) acids, were selected for further evaluation. Signal intensities were normalized to the internal standard nor-valine (TBDMS derivative) and further corrected for the weight of the extracted fecal sample. Quality control was assessed using pooled QC samples analyzed at the beginning and end of the analytical sequence. SCFAs are reported as normalized log_2_-transformed relative signal intensities rather than absolute concentrations.

### 2.6. Statistical Analysis

Statistical analyses were performed using R (version 4.3.2) and Python (version 3.10) software environments. Microbiome and metabolomic data were evaluated using nonparametric statistical tests due to small sample size and non-normal data distribution. Group comparisons were conducted using the Mann–Whitney U test, and results are presented as medians with interquartile ranges unless otherwise stated. Group differences are interpreted together with these descriptive statistics to provide an estimate of effect magnitude alongside statistical significance. A p-value < 0.05 was considered statistically significant. Graphical visualization was performed using appropriate statistical and plotting libraries.

For multivariate analysis of global untargeted metabolomic data, principal component analysis (PCA) was applied as an unsupervised exploratory method. Prior to PCA, metabolite intensities were log-transformed [log(x + 1)] and auto-scaled by mean centering and division by the standard deviation. Missing values in the global metabolomic dataset were imputed using median imputation to preserve the overall variance structure.

Targeted SCFA analyses were processed separately due to their distinct analytical purpose. For SCFAs, missing values were imputed using the half-minimum rule prior to log_2_(x + 1) transformation, reflecting the low-abundance nature of specific analytes and common practice in targeted metabolite quantification workflows. Missingness rates per SCFA (0–21%) are reported in Supplementary Table S2.

## 3. Results

### 3.1. Gut Microbiome Composition

**Figure 1:**
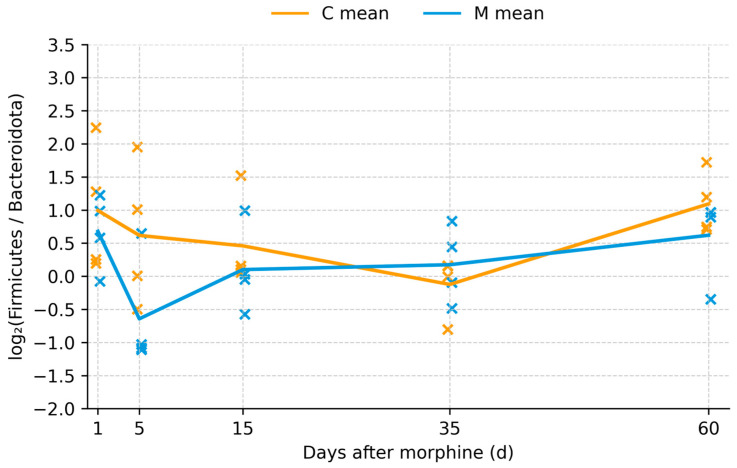
Log_2_(Firmicutes/Bacteroidota) ratio per sample over time. Scatter plot of the log_2_-transformed F/B ratio computed from relative abundances (total sum scaling, pseudocount 1 × 10^−6^) in control (C) and morphine-treated (M) rats at days 1, 5, 15, 35, and 60 after treatment. Individual crosses represent single animals (orange = control, blue = morphine-treated), with control samples slightly offset to the left and morphine samples to the right for clarity. Solid lines indicate group means at each time point. Each point represents an independent fecal sample from a different animal (n = 4 per group per time point).

**Figure 2:**
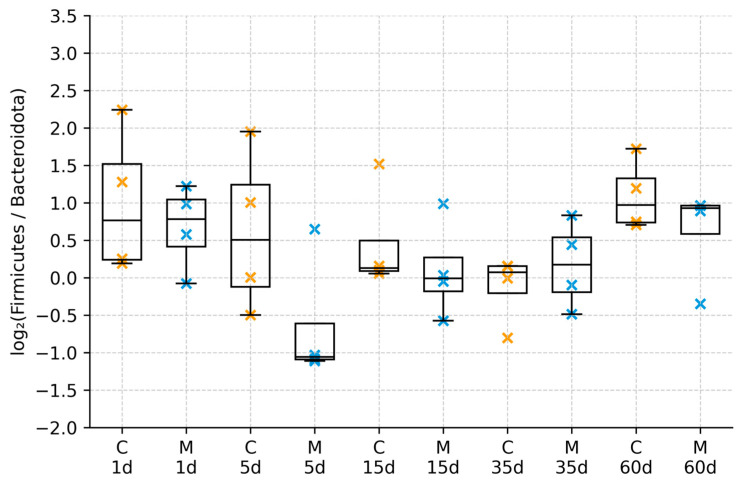
Distribution of log_2_(Firmicutes/Bacteroidota) ratio by group and day. Boxplots of the log_2_ F/B ratio (relative abundances; pseudocount 1 × 10^6^) for control (C) and morphine-treated (M) groups across all time points. Boxes represent the interquartile range (Q1–Q3) with the horizontal line indicating the median; whiskers denote the minimum and maximum values. Individual crosses represent single animals (orange = control, blue = morphine-treated). Each point represents an independent fecal sample from a different animal (n = 4 per group per time point).

Analysis of fecal microbiome composition revealed distinct temporal changes following morphine administration and withdrawal. Shortly after cessation of morphine treatment, alterations in the relative abundance of major bacterial phyla were observed, with increased variability of the Firmicutes/Bacteroidota ratio in morphine-treated animals compared with controls. In particular, shifts in the proportions of Firmicutes and Bacteroidota were detected, as reflected by changes in the Firmicutes/Bacteroidota (F/B) ratio (Figure 1; Supplementary Table S3). At early withdrawal time points, morphine-treated rats exhibited increased variability in F/B ratios relative to controls. Although median F/B ratios differed between groups shortly after treatment cessation, these differences diminished during prolonged withdrawal, and values converged toward those observed in control animals (Figure 2). No persistent long-term differences in F/B ratios were detected.

### 3.2. Alpha Diversity of the Gut Microbiome

**Figure 3:**
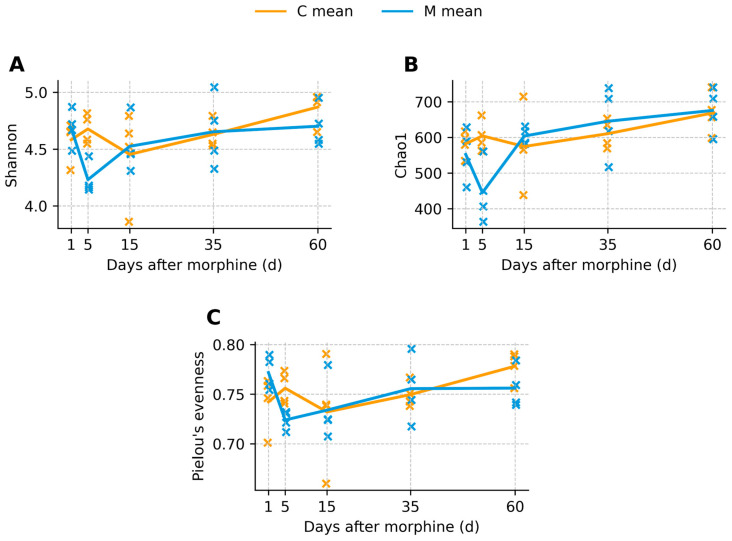
α-diversity per sample over time (scatter): (**A**) Shannon diversity, (**B**) Chao1 richness, and (**C**) Pielou’s evenness in control (C) and morphine-treated (M) rats at days 1, 5, 15, 35, and 60 post-treatment. Individual crosses represent single animals (orange = control, blue = morphine-treated), with control samples slightly offset to the left and morphine samples to the right for clarity. Solid lines indicate group means at each time point. Each point represents an independent fecal sample from a different animal (n = 4 per group per time point).

**Figure 4:**
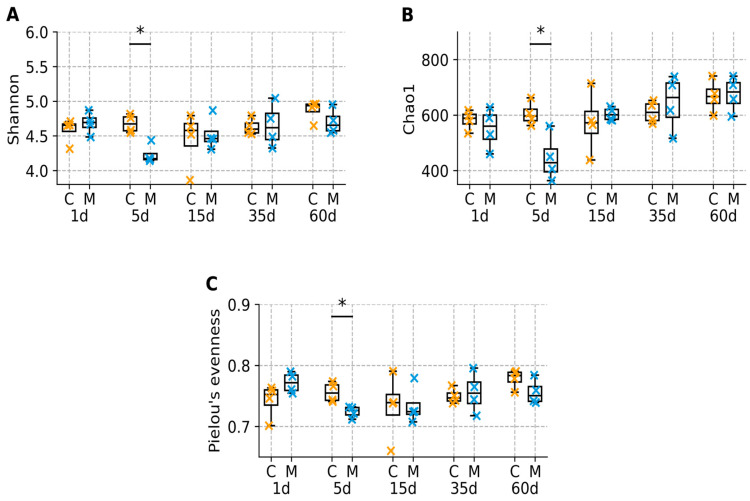
Distribution of α-diversity by group and day (boxplots): (**A**) Shannon diversity, (**B**) Chao1 richness, and (**C**) Pielou’s evenness across time points. Boxes show the interquartile range (Q1–Q3) with the median; whiskers denote the data range. Individual points represent samples from individual animals (control = orange points, morphine = blue points). Each symbol corresponds to one fecal sample from an individual animal. Asterisks above day 5 indicate nominal between-group differences based on two-sided Mann–Whitney tests (*, raw *p* < 0.05). FDR-adjusted q-values are reported in Supplementary Table S4. Each point represents an independent fecal sample from a different animal (n = 4 per group per time point).

Alpha diversity analysis demonstrated a transient reduction in microbial diversity following morphine withdrawal (Figure 3 and Figure 4). At day 5, Shannon diversity, Chao1 richness, and Pielou’s evenness showed nominal between-group differences (raw *p* = 0.029 for each of the three indices; Supplementary Table S4); however, these differences did not remain statistically significant after Benjamini–Hochberg FDR correction (q = 0.145). These trends suggest a transient reduction in microbial diversity shortly after morphine discontinuation, although the magnitude of the differences was moderate and did not remain significant after correction for multiple testing. During prolonged withdrawal, alpha diversity indices in morphine-treated animals were comparable to those observed in control animals, and no persistent group-level differences were detected.

### 3.3. Beta Diversity and Overall Community Differences

**Figure 5:**
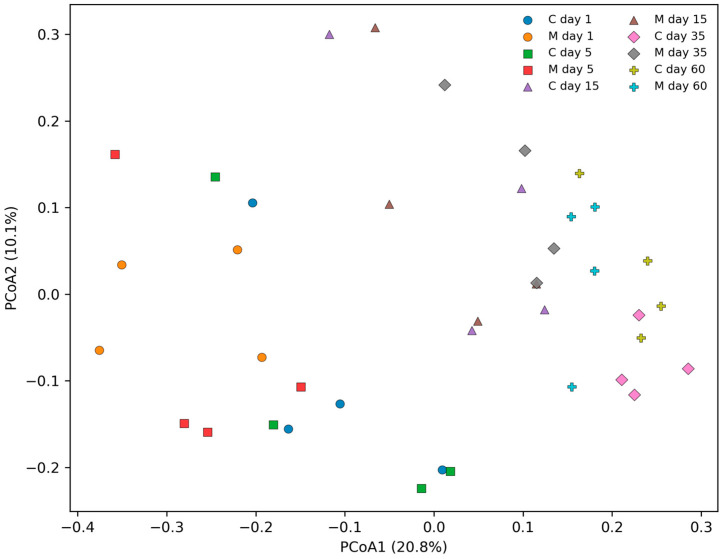
PCoA of gut microbiome composition based on Bray–Curtis dissimilarities. Principal coordinates analysis (PCoA) was performed on rarefied OTU count data (5236 reads per sample). Points represent individual fecal samples from control (C) and morphine-treated (M) animals at days 1, 5, 15, 35, and 60 post-treatment. Axis labels indicate the percentage of variance explained by each coordinate. Overall community differences were assessed using PERMANOVA; dispersion was evaluated using PERMDISP. Each point represents an independent fecal sample from a different animal (n = 4 per group per time point).

Beta diversity analysis based on Bray–Curtis dissimilarities revealed time-dependent shifts in overall community composition and a detectable group effect (Figure 5). PERMANOVA supported statistically detectable effects of group (F = 2.297, *p* = 0.007), time (F = 2.723, *p* = 0.001), and a group × time interaction (F = 1.701, *p* = 0.003), indicating that the magnitude and/or direction of between-group differences varied across time points. Importantly, PERMDISP did not indicate significant heterogeneity of dispersion for group (F = 1.239, *p* = 0.301) or time (F = 1.953, *p* = 0.188), suggesting that PERMANOVA results reflect shifts in centroid locations rather than dispersion-driven artifacts.

### 3.4. Fecal Metabolome

**Figure 6:**
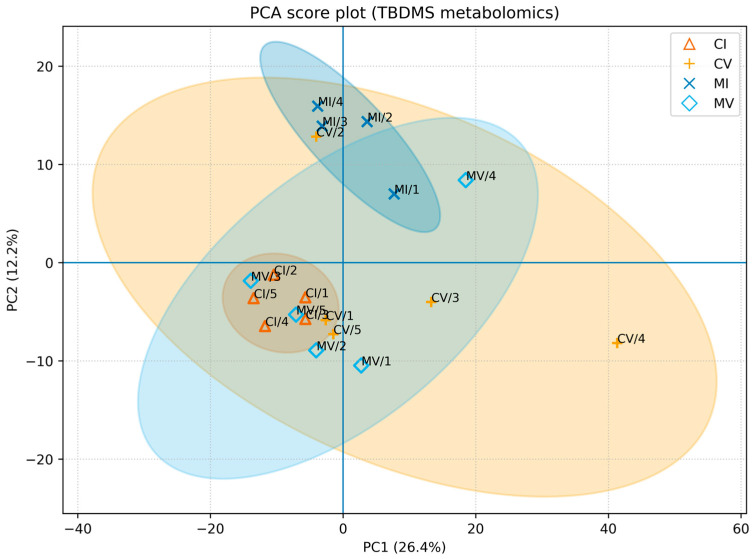
Principal component analysis (PCA) of fecal metabolite profiles. PCA score plot based on log-transformed and auto-scaled global untargeted fecal metabolite intensities obtained by GC×GC-TOF/MS (TBDMS derivatization). Control samples (CI, CV) are shown in orange shades and morphine-treated samples (MI, MV) in blue shades; darker and lighter tones distinguish I (day 1) and V (day 60) subgroups. Ellipses represent 95% confidence intervals for each group. The first two principal components explained 26.4% (PC1) and 12.2% (PC2) of the total variance, respectively. Each point represents an independent fecal sample from a different animal (n = 5 per group).

**Figure 7:**
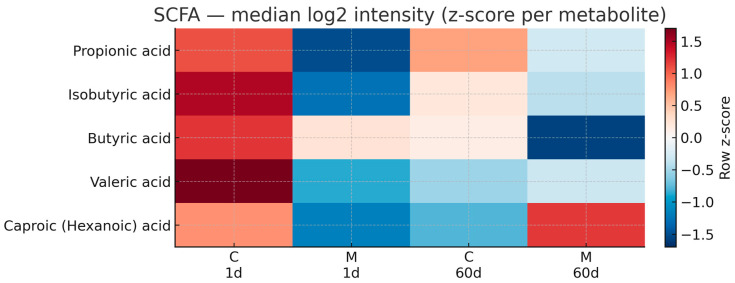
Heat map of short-chain fatty acids (SCFAs) in control (C) and morphine-treated (M) rats. Median log_2_-transformed GC×GC-TOF/MS (TBDMS) intensities are summarized per metabolite across groups and time points; columns are labeled as C/M with the day 1 or 60 on a separate line. Values were z-scored within each metabolite so that 0 represents the within-metabolite mean. The diverging color scale (RdBu_r) is centered at 0 (white), with blue indicating lower-than-average and red higher-than-average values; the color bar reports the row z-score. Missing values were imputed using the half-minimum rule prior to log_2_(x+1) transformation.

**Figure 8:**
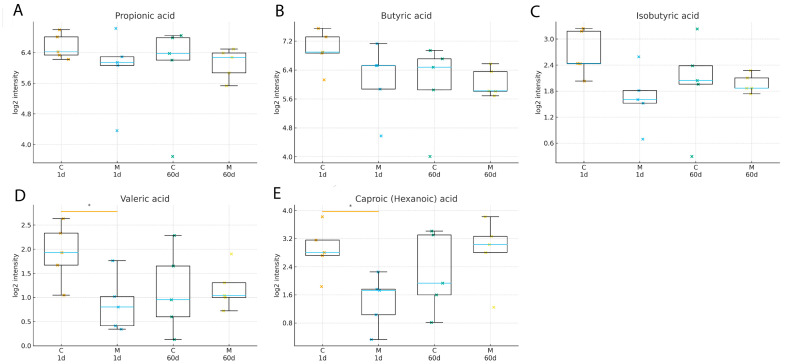
Short-chain fatty acids (SCFAs) in control (C) and morphine-treated (M) rats (box–whisker plots). Box–whisker plots show log_2_-transformed GC×GC-TOF/MS (TBDMS) intensities with individual data points. X-axis labels indicate control (C) and morphine-treated (M) rats at days 1 and 60 after treatment. Boxes represent the interquartile range with the median; whiskers extend to 1.5×IQR. Group differences were tested on each day using two-sided Wilcoxon–Mann–Whitney tests. Asterisks (*) indicate nominal raw *p*-values < 0.05. FDR-adjusted q-values are reported in Supplementary Table S2. Panels: (**A**), propionic acid; (**B**), butyric acid; (**C**), isobutyric acid; (**D**), valeric acid; (**E**), caproic (hexanoic) acid. Each point represents an independent fecal sample from a different animal (n = 5 per group per time point).

To assess global changes in fecal metabolite profiles, unsupervised principal component analysis (PCA) was performed on GC×GC-TOF/MS-derived metabolomic data. The PCA score plot revealed substantial inter-individual variability and partial overlap between control and morphine-treated samples (Figure 6). The first two principal components explained 26.4% (PC1) and 12.2% (PC2) of the total variance, respectively. No clear long-term separation between groups was observed. Targeted analysis of fecal short-chain fatty acids (SCFAs) identified five analytes, including propionic, isobutyric, butyric, valeric, and caproic/hexanoic acids. Shortly after morphine withdrawal, valeric acid (raw *p* = 0.0317) and caproic (hexanoic) acid (raw *p* = 0.0159) showed nominal between-group differences in morphine-treated rats compared with controls (Figure 6; Supplementary Table S2); however, these differences did not remain statistically significant after FDR correction (q = 0.159). These transient differences were primarily driven by lower relative signal intensities of valeric and caproic acids in morphine-treated animals at the early post-discontinuation time point. In contrast, the relative signal intensities of propionic, isobutyric, and butyric acids did not differ significantly between groups at this time point (Figure 7). At later withdrawal stages, no statistically significant differences in SCFA relative signal intensities were detected between control and morphine-treated animals, indicating reduced group-level differences at later post-treatment time points (Figure 7 and Figure 8).

Overall, morphine administration was associated with transient alterations in gut microbiome composition at the group level, including changes in F/B ratios and reduced alpha diversity shortly after treatment cessation. These microbiome alterations were accompanied by short-term changes in selected fecal metabolites, particularly specific SCFAs. During prolonged withdrawal, both microbiome composition and fecal metabolite profiles showed partial normalization, with no persistent long-term differences detected between morphine-treated and control animals.

## 4. Discussion

The present study examined longitudinal changes in gut microbiome composition and fecal metabolomic profiles following morphine administration and prolonged withdrawal in rats. Our results suggest that morphine exposure is associated with transient group-level alterations in gut microbiome composition and selected fecal metabolites during early post-discontinuation phases.

Morphine-induced alterations in the gut microbiome composition observed in this study are consistent with accumulating preclinical and clinical evidence demonstrating that opioids disrupt gut microbial communities [25,26]. Chronic or repeated opioid exposure has been associated with reduced alpha diversity and shifts in the abundance of key bacterial taxa in both humans and rodent models [4,5,7,21]. Similar patterns have been reported in patients receiving opioid agonist therapy, where opioid use was linked to decreased microbial diversity and altered representation of taxa involved in metabolic functions, including butyrate production and bile acid metabolism [27].

Mechanistically, opioid-induced dysbiosis has been linked to impaired gut barrier integrity and altered immune signaling, promoting bacterial translocation and systemic inflammation through pathways involving toll-like receptors [4,6]. These peripheral effects may influence central opioid responses via the gut–brain axis, which has been increasingly implicated in opioid tolerance, withdrawal, and neurobehavioral outcomes [10,21,22]. In line with previous studies, our data suggest that although morphine induces measurable microbiome perturbations during exposure and early withdrawal, group-level differences observed at early post-treatment time points were not detected at later intervals, suggesting that morphine-associated alterations detected shortly after discontinuation are not sustained at the group level over extended time intervals. Beta diversity analysis based on Bray–Curtis dissimilarities further supported time-dependent shifts in overall community composition, with significant effects of group and time detected by PERMANOVA and no evidence of dispersion-driven artifacts (PERMDISP).

Targeted fecal metabolomic analysis revealed transient reductions in valeric and caproic (hexanoic) acids shortly after morphine withdrawal, whereas the relative signal intensities of other SCFAs remained largely unchanged. SCFAs are key microbial-derived metabolites involved in immune regulation, epithelial barrier maintenance, and gut–brain communication [28,29,30]. The selective nature of SCFA alterations observed here suggests targeted modulation of specific microbial metabolic pathways rather than a global disruption of microbial fermentation processes [31,32]. Unsupervised principal component analysis (PCA) of global fecal metabolomic profiles did not demonstrate persistent separation between control and morphine-treated groups during prolonged withdrawal. PCA is inherently an exploratory method that summarizes dominant sources of variance in high-dimensional data and does not necessarily capture subtle but biologically relevant changes in individual metabolites [33,34]. Therefore, the absence of long-term group separation in PCA space does not preclude functionally meaningful metabolic alterations at the level of specific pathways or compounds [35,36]

Recent multi-omics studies have highlighted that changes in microbiome composition do not necessarily translate into large-scale shifts in global metabolomic profiles but may selectively influence specific metabolic pathways of functional relevance [13,37]. The transient and selective metabolomic changes observed in this study are therefore consistent with a model in which opioid exposure induces short-lived functional perturbations that are no longer statistically distinguishable from controls at later post-discontinuation time points.

## 5. Limitations of the Study

A key strength of this study is its time-resolved experimental design, enabling assessment of early and delayed group-level effects of morphine withdrawal on gut microbiome composition and fecal metabolomic profiles. Nevertheless, several limitations should be acknowledged. The relatively small sample size and inter-individual variability limit statistical power, particularly for detecting subtle changes in global metabolomic signatures. Future studies incorporating deeper taxonomic profiling may help identify specific opioid-sensitive microbial taxa. It should also be noted that 16S rRNA gene sequencing provides primarily taxonomic resolution and does not directly capture functional microbial activity; therefore, future studies integrating shotgun metagenomics or metatranscriptomics could further clarify the functional consequences of opioid-associated microbiome perturbations. In addition, while targeted SCFA analysis provides functional insight, untargeted metabolomic approaches or integrated metagenomic and metatranscriptomic analyses may capture additional metabolites or pathways affected by morphine exposure. Furthermore, fecal samples were not consistently collected from the same individual animals at all time points. Therefore, the present study characterizes temporal group-level patterns rather than within-subject longitudinal trajectories, and conclusions regarding intra-individual recovery dynamics cannot be drawn.

Another limitation of the present study is that only male animals were included. This design was chosen to reduce biological variability associated with hormonal fluctuations; however, sex-dependent differences in both gut microbiome composition and opioid responses have been reported. Future studies including both sexes will therefore be important to better understand potential sex-specific microbiome responses to opioid exposure and withdrawal.

## 6. Conclusions

This study demonstrates that repeated morphine administration followed by withdrawal induces transient alterations in both gut microbiome composition and fecal metabolic profiles in rats. Short-term changes were observed in microbial diversity and overall community composition, as indicated by complementary alpha and beta diversity analyses, accompanied by selective alterations in specific fecal metabolites, particularly selected short-chain fatty acids. Importantly, most of the differences observed at early post-discontinuation time points were not detected at later intervals, indicating that morphine-associated alterations are time-dependent at the group level and highlighting the dynamic and reversible nature of opioid-associated perturbations of the gut ecosystem. Unsupervised multivariate analysis of fecal metabolomic profiles did not reveal persistent global separation between control and morphine-treated groups, further supporting the notion that morphine-induced metabolic effects are predominantly temporary at the global level. By combining longitudinal microbiome profiling with fecal metabolomic analysis, this study provides an integrated view of gut ecosystem responses to opioid exposure and withdrawal. Although limited by sample size and inter-individual variability, the findings emphasize the importance of long-term monitoring when assessing drug-induced effects on host–microbiome interactions. Future studies employing larger cohorts and expanded functional analyses will be essential to clarify the mechanistic links between opioid exposure, gut microbial metabolism, and host physiology.

## Data Availability

The original contributions presented in this study are included in the article/Supplementary Materials. Further inquiries can be directed to the corresponding author. Raw 16S rRNA gene sequencing data have been deposited in the NCBI Sequence Read Archive (SRA) under BioProject accession PRJNA1433795. The metabolomics dataset generated in this study has been deposited in the MetaboLights repository under accession number REQ20260224217241.

## Supplementary Materials

The following supporting information can be downloaded at: https://www.mdpi.com/article/10.3390/biom16030460/s1, Figure S1: Rarefaction curves; Table S1: Sample allocation; Table S2: Targeted short-chain fatty acids (SCFAs) in rat fecal extracts; Table S3: Firmicutes/Bacteroidota ratio; Table S4: Alpha diversity by group and day.
