# Supplementary material for: Longitudinal Analysis of Rat Gut Microbiome Composition and Fecal Metabolism Markers Following Prolonged Morphine Exposure
Source: Biomolecules. 2026 Mar 18;16(3):460. doi: 10.3390/biom16030460 (PMC13023539; doi:10.3390/biom16030460)
Supplement: Supplementary file 1 [file biomolecules-16-00460-s001.zip › biomolecules-4166283-supplementary.pdf]

Supplementary Materials

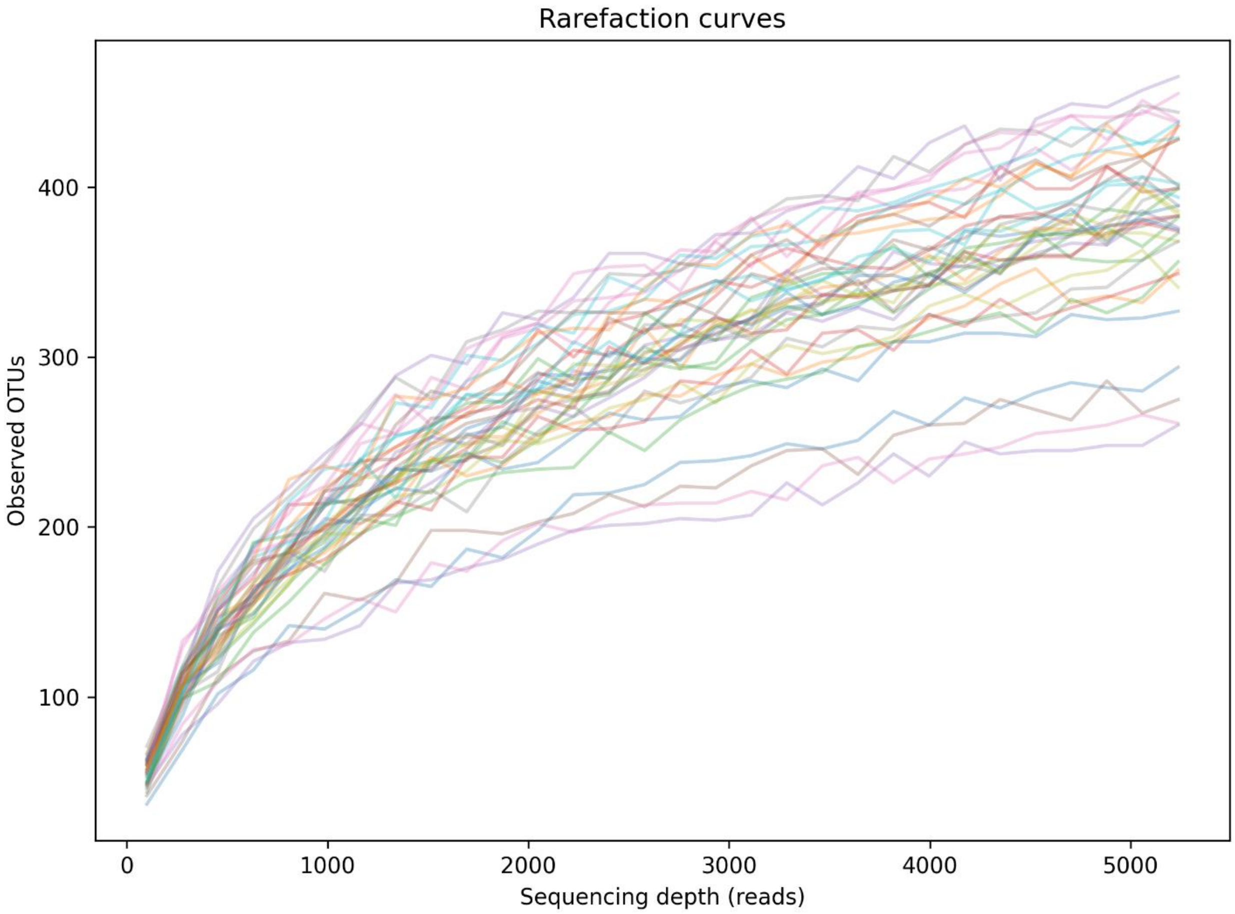

Supplementary Figure S1. Rarefaction curves.

Rarefaction curves depicting the observed OTU richness relative to sequencing depth after subsampling without replacement. All samples demonstrate progressive saturation of diversity prior to the applied rarefaction depth of 5,236 reads.

Supplementary Table S1. Sample allocation and final sample sizes per analytical platform

Analysis	Time points analyzed	Animals available per group	Final n per group per time point	Exclusions	Reason for exclusions
16S rRNA gene sequencing	Days 1, 5, 15, 35, 60	5	4	0	Not applicable (predefined analytical allocation)
Targeted SCFA analysis	Days 1, 60	5	5	0	Not applicable

Five animals per group were available at each predefined time point. For 16S rRNA sequencing, four animals per group were included to enable coverage of multiple withdrawal intervals within available sequencing capacity. For targeted SCFA analysis, five animals per group were analyzed at selected early and late time points. No samples were excluded due to sequencing or metabolomic quality control failure.

Supplementary Table S2. Short-chain fatty acids (SCFAs)

Metabolite	Day	n_C	median_C	IQR_C	n_M	median_M	IQR_M	p_value	q-value (BH-FDR)
Butyric acid	1	5	6.89	0.45	5	6.52	0.66	0.1508	0.377
Butyric acid	60	5	6.48	0.86	5	5.82	0.55	0.4206	0.601
Caproic (Hexanoic) acid	1	5	2.81	0.44	5	1.73	0.73	0.0159	0.159
Caproic (Hexanoic) acid	60	5	1.93	1.71	5	3.03	0.46	0.6905	0.690
Isobutyric acid	1	5	2.43	0.75	5	1.6	0.29	0.0556	0.185
Isobutyric acid	60	5	2.04	0.42	5	1.87	0.24	0.5476	0.684
Propionic acid	1	5	6.42	0.47	5	6.14	0.22	0.2222	0.444
Propionic acid	60	5	6.38	0.58	5	6.28	0.52	0.6905	0.690
Valeric acid	1	5	1.93	0.66	5	0.8	0.61	0.0317	0.159
Valeric acid	60	5	0.95	1.05	5	1.04	0.3	0.6905	0.690

Peak intensities (GC–MS, TBDMS derivatization) were processed by half-minimum imputation (per metabolite) and $\log_2(x+1)$ transformation. Results are reported for control (C) and morphine-treated (M) groups at day 1 (1d) and day 60 (60d).

Sheet "S3_SCFAs_summary" – per metabolite, day, and group: n, median [IQR], and mean ± SD ($\log_2$ scale).

Sheet "S3_CvsM_pvalues" – per metabolite and day: n_C, median_C [IQR_C], n_M, median_M [IQR_M], and the two-sided Wilcoxon–Mann–Whitney tests. P-values were adjusted for multiple comparisons using the Benjamini–Hochberg false discovery rate (FDR) procedure across all SCFA comparisons (10 tests). Both raw p-values and FDR-adjusted q-values are reported.

Abbreviations: IQR, interquartile range (Q3–Q1); SD, standard deviation. Values reflect relative signal intensities on the $\log_2$ scale.

Missingness per metabolite prior to half-minimum imputation ranged from 0% to 21% (propionic acid 15.0%, isobutyric acid 21.0%, butyric acid 5.0%, valeric acid 0%, caproic/hexanoic acid 6.25%).

Supplementary Table S3. Firmicutes/Bacteroidetes ratios

Group	Day	n	median_log2	mean_log2	sd_log2	Q1	Q3
C	1	4	0.7674523207417059	0.9930873223912164	0.9712580775217832	0.2400624255411973	1.520477217591725
C	5	4	0.5064594380818933	0.6171999855725927	1.087368067236472	-0.1196347997071815	1.243294223361668
C	15	4	0.129601149874222	0.4594806347723928	0.7087663181125472	0.09056883925245755	0.4985129453941571
C	35	4	0.07432776767433287	-0.1234002742129605	0.4588414393807826	-0.2043753425467472	0.1553028360081196
C	60	4	0.9717007753635168	1.09321489813972	0.4741361052018795	0.7378482432223683	1.327067430280868
M	1	4	0.7831590304224769	0.6790094641752846	0.5689175251491791	0.4165017162907396	1.045666778307022
M	5	4	-1.056238186164557	-0.6428237202073102	0.8618437385645038	-1.088875815522025	-0.6101860908498424
M	15	4	-0.007390688548990672	0.1007879121600197	0.6512642788872456	-0.179580983887899	0.272978207498928
M	35	4	0.1748957358882059	0.1744670300235363	0.581273276737083	-0.1922402259943023	0.5416029919060444
M	60	4	0.9290703437091563	0.6199903776667572	0.6447162197273566	0.5842598477738433	0.9648008736020702

Summary statistics of the Firmicutes/Bacteroidota (F/B) ratio calculated from relative abundances (total sum scaling, pseudocount 1e-6) in control (C) and morphine-treated (M) rats at days 1, 5, 15, 35, and 60 post-treatment. Values are reported as the number of animals (n), median with interquartile range (IQR), mean, and standard deviation (SD) of the $\log_2$-transformed F/B ratio.

Supplementary Table S4. Alpha diversity indices

Index	Day	n_C	C median (IQR)	C mean	C SD	n_M	M median (IQR)	M mean	M SD	p-value (C vs M)	q-value (BH-FDR)
Chao1	1	4	589 (568–603)	582	35	4	560 (513–600)	552	74	0.686	0.886
Chao1	5	4	596 (580–621)	604	43	4	428 (396–478)	445	85	0.029	0.145
Chao1	15	4	572 (534–613)	574	113	4	601 (584–621)	604	24	0.343	0.643
Chao1	35	4	610 (580–640)	610	40	4	663 (592–716)	645	100	0.686	0.886
Chao1	60	4	667 (642–693)	668	59	4	683 (642–717)	676	64	1.000	1.000
Pielou’s evenness	1	4	0.752 (0.735–0.760)	0.742	0.028	4	0.771 (0.759–0.784)	0.772	0.017	0.200	0.375
Pielou’s evenness	5	4	0.755 (0.743–0.768)	0.756	0.016	4	0.726 (0.719–0.731)	0.724	0.009	0.029	0.145
Pielou’s evenness	15	4	0.739 (0.719–0.752)	0.732	0.054	4	0.724 (0.720–0.738)	0.734	0.031	0.686	0.886
Pielou’s evenness	35	4	0.747 (0.742–0.755)	0.75	0.013	4	0.755 (0.738–0.773)	0.756	0.033	0.886	0.951

Pielou's evenness	60	4	0.783 (0.773–0.789)	0.778	0.016	4	0.751 (0.741–0.765)	0.756	0.021	0.200	0.375
Shannon	1	4	4.654 (4.565–4.671)	4.583	0.18	4	4.694 (4.622–4.758)	4.686	0.159	0.343	0.643
Shannon	5	4	4.672 (4.575–4.774)	4.677	0.131	4	4.172 (4.159–4.244)	4.231	0.138	0.029	0.145
Shannon	15	4	4.580 (4.356–4.677)	4.453	0.41	4	4.461 (4.420–4.566)	4.525	0.239	0.886	0.951
Shannon	35	4	4.598 (4.544–4.684)	4.63	0.121	4	4.619 (4.447–4.825)	4.652	0.315	0.886	0.951
Shannon	60	4	4.935 (4.848–4.957)	4.87	0.148	4	4.653 (4.573–4.781)	4.701	0.184	0.200	0.375

Per-sample α-diversity indices (Shannon, Chao1, Pielou's evenness) were calculated from the OTU count table after rarefaction to a common sequencing depth. For each day (1, 5, 15, 35, 60), values are summarized for control (C) and morphine-treated (M) rats as median (IQR) and mean ± SD alongside the sample size (n). Between-group differences at each day were assessed with two-sided Mann–Whitney U tests (p-value shown in the rightmost column). P-values were adjusted using Benjamini–Hochberg FDR across all alpha-diversity tests (15 comparisons); both raw p-values and FDR-adjusted q-values are reported.
